# Development of Pectin-Based Aerogels with Several Excellent Properties for the Adsorption of Pb^2+^

**DOI:** 10.3390/foods10123127

**Published:** 2021-12-16

**Authors:** Risi Wang, Ya Li, Xixiang Shuai, Jun Chen, Ruihong Liang, Chengmei Liu

**Affiliations:** 1 State Key Laboratory of Food Science and Technology, Nanchang University, Nanchang 330047, China; ncuskwangrisi@163.com (R.W.); shuaixixiang1989@163.com (X.S.); chen-jun1986@hotmail.com (J.C.); liangruihong@ncu.edu.cn (R.L.); 2 South Subtropical Crop Research Institute, China Academy of Tropical Agricultural Sciences, Zhanjiang 524091, China; liya1995ncu@163.com

**Keywords:** pectin, PEI, EGDE, aerogel, Pb^2+^, adsorption

## Abstract

Traditional aerogels lack specific functional groups for the adsorption of Pb^2+^, which results in a low adsorption capacity and limits the application scope. Novel porous pectin-based aerogels (PPEAs) were prepared by incorporating polyethylenimine (PEI) using ethylene glycol diglycidyl ether (EGDE) as a cross-linker for the removal of Pb^2+^ from water. The cross-linking mechanism, morphology, mechanical strength, thermal stability, adsorption properties, and mechanism of the aerogels were investigated. The aerogels possessed several desirable features, such as a large maximum Pb^2+^ adsorption capacity (373.7 mg/g, tested at pH 5.0), ultralight (as low as 63.4 mg/cm^3^), high mechanical strength (stress above 0.24 MPa at 50% strain), and easy recyclability. Meanwhile, the equilibrium adsorption data was well described by the Langmuir–Freundlich (Sips) model and the kinetic adsorption process was well fitted using the pseudo-second-order model. The donor groups, such as -NH_2_, and oxygen-containing functional groups were responsible for the Pb^2+^ adsorption, which was confirmed by the FTIR and XPS analysis. The excellent characteristics mean that PPEAs are highly effective adsorbents in the remediation of lead-containing wastewater.

## 1. Introduction

Lead pollution, which is one of the most serious environmental problems, mainly comes from mining, batteries, glass manufacturing, metallurgy, printing, and wastewater from smelters [1]. After entering the water, Pb^2+^ is difficult to degrade and will mainly be transferred and transformed through chelation, colloid formation, adsorption, resolution, etc., causing accumulation in organisms through the biological amplification effect of the food chain [2], thus affecting the normal growth of animals and pose a threat to human health [3]. In addition, Pb^2+^ is a plant stressor that affects the growth of plants [4]. In China, the limit for lead in drinking water is 0.01 mg/L (GB 5749-2006). The excessive intake of Pb^2+^ mainly harms the central nervous system, digestive system, reproductive system, liver, kidney, and bone marrow hematopoietic function of the human body [5,6].

At present, the methods for removing Pb^2+^ from wastewater include biological treatment, chemical precipitation, coagulation, ion exchange, membrane filtration, and adsorption [7,8]. Among those methods, adsorption is considered to be the most environmentally friendly and effective method for the treatment of heavy metal wastewater since it has good repeatability, is simple, has a high treatment efficiency, and is a mature and stable process that is suitable for large-scale popularization and applications [9,10].

Aerogel is a kind of porous solid material with a highly interpenetrated structure. Due to its unique properties, such as ultra-low density, high porosity, shape variability, and ease of separation and recovery without secondary pollution, aerogels have attracted significant attention regarding green and efficient wastewater treatment [11]. Compared with hydrogels that are used as adsorbents, aerogels have obvious advantages, such as being ultralight and moisture free, which make aerogels easy to transport and store. However, due to the lack of specific functional groups on the surface, a single aerogel exhibits a low adsorption capacity and cannot directly adsorb heavy metals. These shortcomings greatly limit the application scope of aerogels. Some modified aerogels were created, such as MOF aerogel, which has attracted research interest because of its outstanding flexibility, interesting structures, and excellent adsorption characteristics [12]. However, the MOF aerogel contained some heavy metal elements, which results in relatively poor biocompatibility. Other modified aerogels with good biocompatibility and biodegradability are urgently needed for further study.

Pectin is a kind of natural anionic polysaccharide that is different from starch and cellulose. Pectin contains many -COOH groups and exhibits a strong heavy metal removal capacity. In addition, pectin also shows excellent properties, such as good biocompatibility, biodegradability, and high abundance. Recently, pectin itself, as well as pectin-based hydrogels, were developed for the adsorption of Pb^2+^ from wastewater [13]. Although some studies reported the unsatisfactory effect of pure pectin on the adsorption of heavy metals [14,15], the adsorption performance of pectin may be significantly improved by the fabrication of pectin-based aerogels because the aerogels have multitudinous advantages, as we described above. However, only a few studies of pectin-based aerogels were reported and they are tentatively used in the fields of drug delivery and thermal insulation [16,17]. Very few works were carried out on heavy metal adsorption by pectin-based aerogels.

In this study, pectin was cross-linked with polyethylenimine (PEI), which is a reagent that is widely used in heavy metal adsorption because it consists of a great number of amine groups [18,19], to prepare porous pectin-based aerogels (PPEAs). Different contents of ethylene glycol diglycidyl ether (EGDE) were used as the cross-linking agent to regulate the degree of modification (*DM*) of the PPEAs. The physicochemical properties, adsorption behaviors, and adsorption mechanism of the PPEAs toward Pb^2+^ were characterized. The information obtained will expand the application of pectin-based aerogels and provide novel materials for the treatment of lead-containing wastewater.

## 2. Materials and Methods

### 2.1. Materials

Citrus pectin (type H121, galacturonic acid content of 82.29%, molecular weight of 527 kDa, esterification degree of 58.48%) was supplied by the general agent of CPkelco (Shanghai, China); the parameters were measured in our previous study [20]. Polyethylenimine (PEI, molecular weight of 10 kDa), ethylene glycol diglycidyl ether (EGDE), sodium hydroxide, hydrochloric acid, nitric acid, and Pb(NO_3_)_2_ were purchased from Aladdin Reagent Company (Shanghai, China). All reagents were of analytical reagent grade and used as received. All aqueous solutions were prepared with deionized water from a Milli-Q system (Millipore, Billerica, MA, USA).

### 2.2. Preparation of PPEA

**Figure 1:**
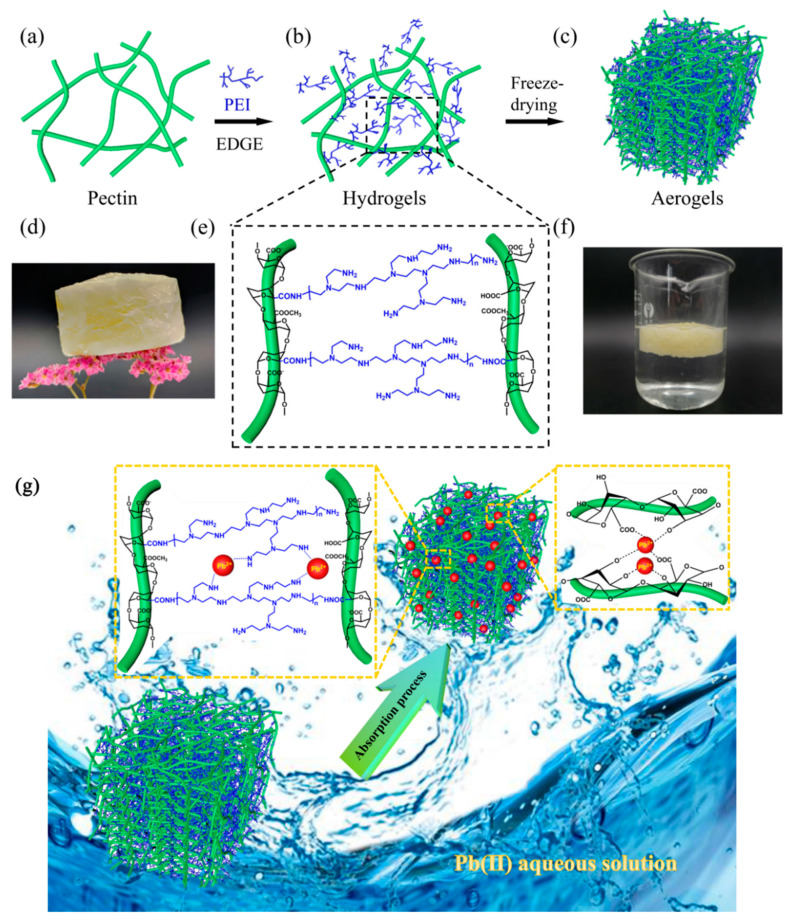
Illustration of the preparation (**a**–**c**) and chemical structure (**e**) of the PPEAs. The PPEAs standing on a dried flower (**d**) and floating on water (**f**). The adsorption mechanism of capturing Pb^2+^ using PPEAs (**g**).

The preparation of the PPEAs is briefly depicted in Figure 1a–c and the details are as follows: 3 g of pectin was dissolved in 100 mL deionized water and PEI was added into the solution with the mass ratio of pectin to PEI of 2:3. The mixture was stirred continuously to form a uniform aqueous solution. The cross-linking agent EGDE was added with different mass fractions (0.05, 0.1, 0.2, 0.3%), and the bubbles were eliminated using an ultrasonic treatment. The mixture was incubated in a 60 °C water bath to form a gel. After that, the prepared gel was soaked in deionized water for 3 days and the water was replaced every 8 h to remove the unreacted impurities. Finally, the dried aerogel samples were obtained by freeze-drying and named PPEA_0.05_, PPEA_0.1_, PPEA_0.2_, and PPEA_0.3_ according to their respective mass fractions.

### 2.3. Dynamic Rheological Study

The dynamic rheological characteristics of the pectin, PEI, pectin/PEI, and pectin/PEI/EGED solutions during the hydrogel formation were investigated using an ARES-G2 rheometer (TA Instruments, New Castle, DE, USA). To guarantee that all measurements were taken in the linear viscoelastic region, amplitude sweep tests were first performed (data not shown) using a parallel plate geometry (40-mm diameter) with a gap size of 1 mm at a constant frequency of 1 Hz when the strain γ ranged from 0.01 to 100%. After that, the change in the storage modulus (G′) and loss modulus (G″) over time was carried out at a constant frequency of 1 Hz and strain amplitude of 0.5% within the linear viscoelastic region (0.1–1.5%). The samples were covered with a thin layer of low-viscosity silicone oil to prevent the evaporation of water during the measurement.

### 2.4. Characterization of PPEAs

#### 2.4.1. Elemental Analysis

The elemental analysis was carried out with an elemental analyzer (Vario Micro cube Elementar, Langenselbold, Germany). The degree of modification (*DM*), which is defined as the number of PEI molecules attached per 100 galacturonic acid molecules, was calculated using the following method.

First, supposing that *X* PEI molecules were attached to *Y* pectin molecules, the content nitrogen element in PEI ($m_{N}$) is

(1) $$m_{N}=\frac{{14\;M}_{\mathrm{PEI}}\times X}{M_{\mathrm{EI}}{\times R}_{0}}$$

where $R_{0}$ is Avogadro’s constant and $M_{\mathrm{PEI}}$ and $M_{\mathrm{EI}}\;$ are the molecular weights of PEI and ethylenimine, respectively.

The carbon content in pectin ($m_{C2}$) is

(2) $$m_{C2}=m_{D}-m_{C1}=m_{D}-\frac{2\times 12}{14}m_{N}$$

where $m_{D}$ is the amount of the carbon element of PPEA as determined by the elemental analyzer and $m_{C1}$ is the carbon content of PEI.

At the same time:

(3) $$m_{C2}=\frac{{Y\times M}_{\mathrm{pectin}}\times 6\times 12}{M_{\mathrm{GalA}}{\times R}_{0}}$$

where $M_{pectin}$ and $M_{GalA}$ are the molecular weights of pectin and galacturonic acid, respectively. 

Therefore, the number of PEI molecules that are attached per pectin (*N*) molecule is

(4) $$N=\frac{{24\;m}_{N}}{{14\;m}_{C}-{24\;m}_{N}}\times \frac{{72\;M}_{\mathrm{EI}}{\times M}_{\mathrm{pectin}}}{{14\;M}_{\mathrm{PEI}}{\times M}_{\mathrm{GalA}}}$$

and the degree of modification (*DM*) was calculated as follows:

(5) $$\mathrm{DM}(\%)=\frac{{N\times M}_{\mathrm{GalA}}}{M_{\mathrm{pectin}}{\times C}_{\mathrm{GalA}}}\times 100=\frac{{24\;m}_{N}}{{14\;m}_{C}-{24\;m}_{N}}\times \frac{{72\;M}_{\mathrm{EI}}}{{14\;M}_{\mathrm{PEI}}{\times C}_{\mathrm{GalA}}}\times 100$$

where $C_{GalA}$, which is the galacturonic acid content of pectin, is 0.8229 g/g.

#### 2.4.2. Other Characterizations

Fourier transform infrared (FTIR) spectra were examined using a Nicolet 5700 spectrometer (Thermo Fisher Scientific, Madison, WI, USA) in the range from 4000 to 400 cm^−1^ with a resolution of 4 cm^−1^. The morphologies of the samples were measured using SEM (JEOL JSM-6701 F Instrument, Tokyo, Japan). The compression test was carried out using a TA-XT2i Texture Analyzer (Stable Micro Systems, Surrey, UK). The aerogels were deformed via compression at a constant speed of 0.01 mm/s to a strain of up to 50% using a cylindrical probe P/36R. The water contact angle was measured using an OCA 25 tester (Dataphysics Instruments, GmbH, Filderstadt, Germany). The thermal properties were analyzed using thermogravimetric analysis (TGA-50, Shimadzu, Tokyo, Japan) in the temperature range 30–600 °C at a heating rate of 20 °C/min under an Ar atmosphere. X-ray photoelectron spectroscopy (ESCALAB250Xi, Thermo Fisher Scientific, Madison, WI, USA) was conducted to analyze the surface chemical composition of the samples before and after adsorption.

### 2.5. Batch Adsorption Experiments

To evaluate the adsorption performance of the samples, a series of adsorption experiments was conducted by changing the pH value (2.0–6.0), contact time (2–600 min), and initial Pb^2+^ concentration (20–600 mg/L). For the regeneration study, the PPEAs were regenerated using a 0.1 mol/L HNO_3_ solution. After the adsorption, the clear supernatant could easily realize solid–liquid separation via salvaging, and the concentrations of Pb^2+^ in the supernatant were monitored using an atomic absorption spectrophotometer (AAS, A3AFG-12, Puxi, Beijing, China). The removal rate *R* (%) and the adsorption capacity *q_e_* (mg/g) for Pb^2+^ were calculated as follows:

(6) $$R=\frac{C_{0}-C_{e}}{C_{0}}\times 100$$

(7) $$q_{e}=\frac{V(C_{0}-C_{e})}{m}$$

where *C*_0_ and *C_e_* (mg/L) are the initial and equilibrium concentrations of Pb^2+^ solution, respectively. *V* (L) is the volume of the solution and *m* (g) is the mass of the PPEA.

## 3. Results and Discussion

### 3.1. Dynamic Rheological Properties of PPEA Hydrogels

**Figure 2:**
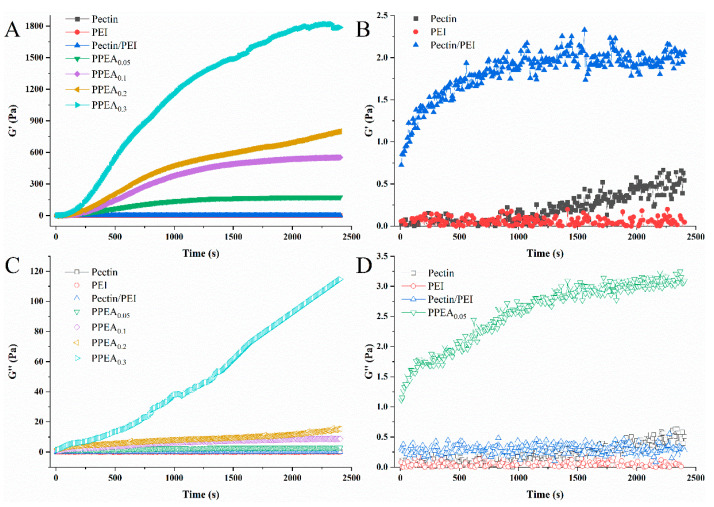
(**A**) Time dependence of the elastic modulus (G′), (**B**) partial enlargement of (**A**), (**C**) loss modulus (G″), and (**D**) partial enlargement of (**C**) for the pectin, PEI, pectin/PEI, and pectin/PEI/EGDE solutions incubated at 60 °C.

The properties of aerogels are directly affected by their corresponding hydrogels [21]. Therefore, dynamic time-sweep oscillatory analysis of the pectin, PEI, pectin/PEI, and pectin/PEI/EGDE solutions was undertaken to monitor the formation of the hydrogel, and the results are shown in Figure 2. Under the conditions of this experiment, the pectin itself could not form gels in water. Low G′ and G″ values were observed in both the pure pectin and PEI solutions, just like those of most polymer solutions. However, when the pectin was mixed with PEI, the G′ was somewhat increased, with G′ > G″, indicating the formation of a weak gel network, probably due to the formation of intermolecular hydrogen bonds between -COOH, the -OH of pectin, and the -NH_2_ of PEI. With the incorporation of EGDE, covalent polymerization between pectin and PEI occurred (Figure 1b,e) and formed a strong cross-linking network, resulting in the G′ of the PPEA hydrogel increasing quickly (Figure 2A,B). The cross-linking structure influenced the mechanical property, morphology, and Pb^2+^ adsorption behavior of the PPEAs, as will be confirmed in the next section.

### 3.2. Characterization of PPEAs

#### 3.2.1. Degree of Modification of PPEAs

The *DM*s of the PPEAs were calculated from the results of elemental analysis according to the Equations (1)–(5) and are listed in Table 1. It was found that *DM* increased with the amount of EGDE. When EGDE was added at 0.05%, 0.1%, 0.2%, and 0.3%, *DM* was 4.66 ± 0.03, 6.23 ± 0.03, 6.74 ± 0.05, and 7.67 ± 0.08%, respectively. It was found that a high EGDE concentration showed a better modification performance because when the amount of PEI was sufficient, more EGDE promoted the amidation reaction. Having more PEI attached to a pectin molecule may be conducive to heavy metal adsorption because PEI contains abundant amine groups, which can interact with heavy metals through chelation [22,23].

#### 3.2.2. FTIR before Adsorption

**Figure 3:**
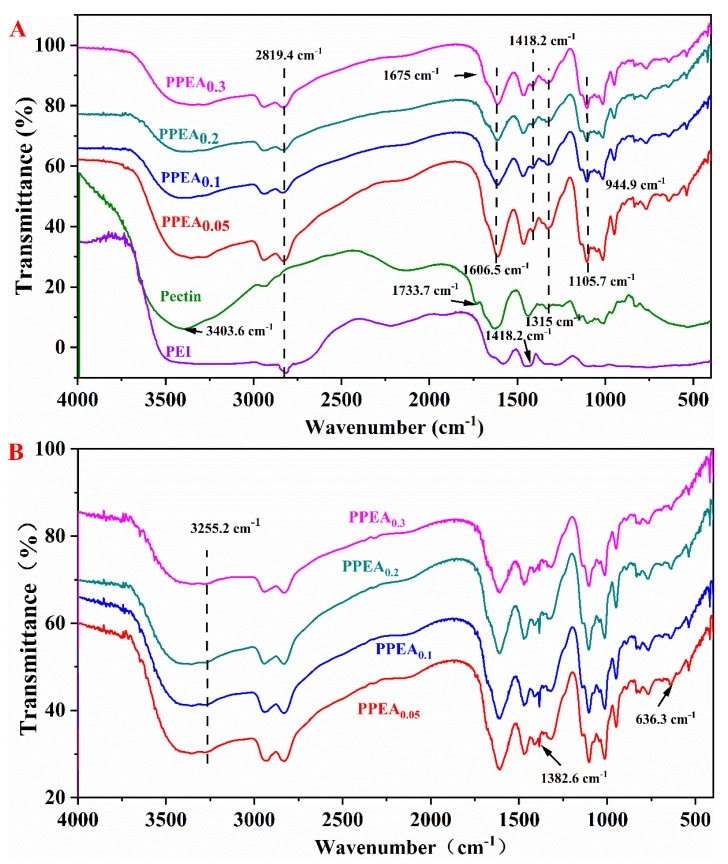
FTIR spectra of the PPEAs before (**A**) and after (**B**) the Pb^2+^ adsorption. The FTIR spectra of pectin and PEI are also provided in part A for comparison.

The covalent cross-linking between pectin and PEI was supported by the FTIR results (Figure 3A). Compared with pectin, the absorption band of the PPEAs around 3400 cm^−1^ (corresponding to the stretching vibration of the -OH group and hydrogen bonding) became wider and stronger, which was caused by the increased number of amine groups in the structure [24]. The absorption peaks at 2842 cm^−1^ were assigned to the symmetric vibration of the -CH_2_ groups in the PEI. The peaks at around 1633 cm^−1^ were also broadened, which were assigned to the overlap of the bending vibration of -NH_2_ group and asymmetric stretching of the carboxylic C=O double bond of new amide linkages and carboxyl. The peak at 1418 cm^−1^ was attributed to the N-H bending vibration peak. The PEI sample had an obvious peak due to the N-H bending vibration. This particular peak in the PPEAs also existed because of the addition of PEI. The stretching vibration peak appeared at 1105.7 cm^−1^, indicating the formation of -CONH- linkages [25]. The peak at 1315 cm^−1^ was the C-O vibration peak. The peak could be observed at all the PPEAs and pectin samples but not in the PEI because the C-O functionalities did not exist in the PEI. A peak at 1733.7 cm^−1^ for the C=O bond of carboxyl groups and their esters disappeared, which also confirmed the reaction between the -COOH of the pectin and the -NH_2_ of the PEI. All the above results indicated that PEI was indeed introduced into the PPEAs through covalently cross-linking the -COO^−^ of pectin with the amido groups of PEI to form -CONH- linkages, which directly contributed to the development of the aerogel [26].

#### 3.2.3. Morphology

The optical image of PPEA_0.3_, as a representative aerogel, is shown in Figure 1d. The PPEA_0.3_ is stood on a dried flower, indicating its ultralight feature. The densities (ρ) of the four PPEAs were calculated using the formula ρ = m/v, where m and v represent the mass and the volume of the PPEA, respectively. It was found that the densities of PPEA_0.05_, PPEA_0.1_, PPEA_0.2_, and PPEA_0.3_ were 107.2, 104.8, 78.4, and 63.4 mg/cm^3^, respectively. These densities were lower than those of many reported polysaccharide-based aerogels, such as chitin-based aerogel (120~220 mg/cm^3^) [27] and alginate aerogel (130 mg/cm^3^) [28]. The low density of the PPEAs endowed them the ability to float on the water’s surface for Pb^2+^ adsorption (Figure 1f) such that they had the advantages of easy removal and reuse.

**Figure 4:**
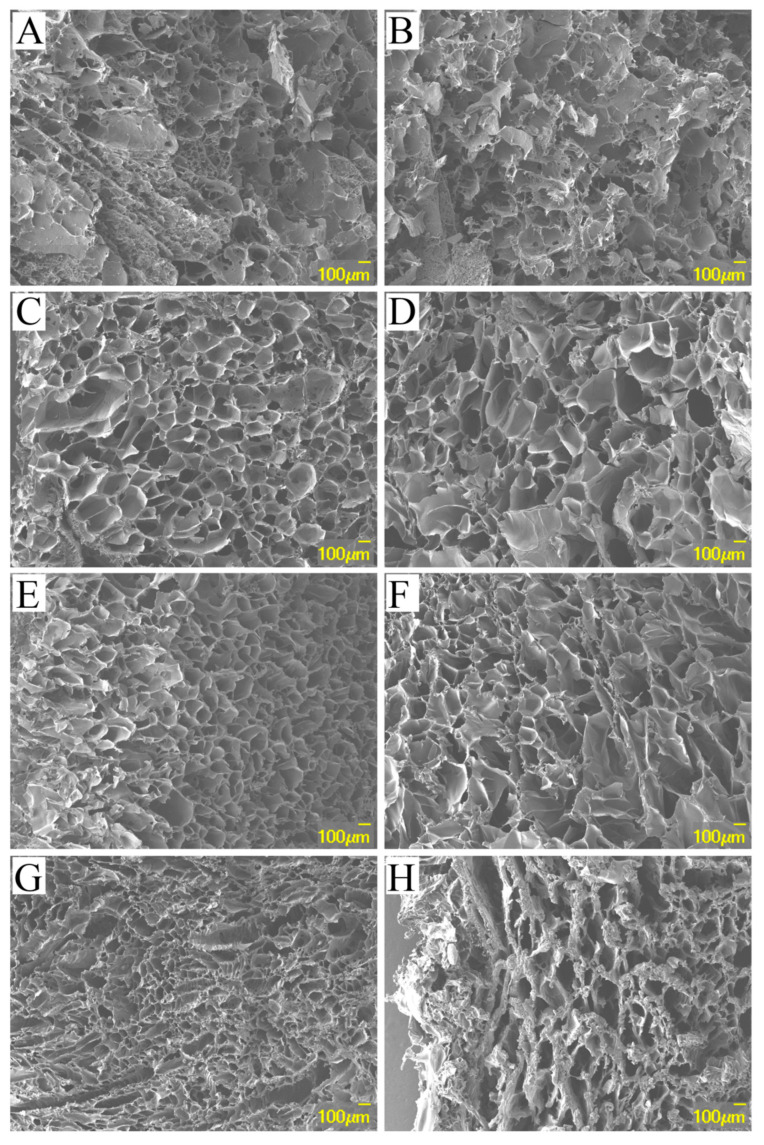
The SEM images of PPEA_0.05_ (**A**,**B**), PPEA_0.1_ (**C**,**D**), PPEA_0.2_ (**E**,**F**), and PPEA_0.3_ (**G**,**H**) before (**A**,**C**,**E**,**G**) and after (**B**,**D**,**F**,**H**) the Pb^2+^ adsorption.

The detailed morphologies of the PPEAs were observed using SEM, where the results showed that the aerogels were typical macroporous materials with 3D network structures. The morphology of the PPEAs varied greatly. From Figure 4A, it was observed that PPEA_0.05_ was composed of a fibrillated and lamellar structure in the network, where the fibril structure was dominant. With the increase in the EGDE amount, the structure of the aerogels became more regular and the pores became smaller and denser. The morphologies of PPEA_0.1_ and PPEA_0.02_ appeared like a “honeycomb” (Figure 4C,E), while the morphology of PPEA_0.3_ looked like a “sponge” (Figure 4G). The morphology may be associated with the cross-linking effect between pectin and PEI that was induced by the EGDE, where the EGDE played a critical role in stabilizing the aerogel skeleton and maintaining the structural integrity [29]. The open porous structure may allow the solution to easily enter the interior of the aerogel, thus affecting the adsorption efficiency.

#### 3.2.4. Mechanical Properties and Wettability

**Figure 5:**
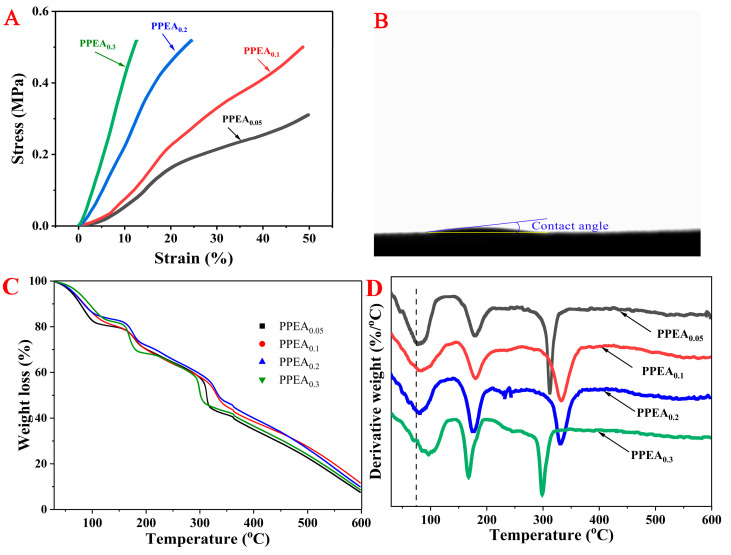
Compressive stress–strain curves of one compress–release cycle of PPEAs with strain up to 50% (**A**). Water contact angle of PPEA_0.03_ as a representative (**B**). TGA patterns (**C**) and the corresponding derivative weight curves (**D**) of the PPEAs.

Appropriate mechanical properties are vital for the practical application of aerogels. The stress–strain mechanical properties are presented in Figure 5A. At the beginning of the compression test, the slope of the stress–strain curve was very large, and the slope of the curve increased with the amount of EGDE. The stress of PPEA_0.3_ and PPEA_0.2_ reached the maximum loading force of the texture analyzer (7000 N) at the strains of 12.7 and 24.6%, respectively. In addition, the stresses of PPEA_0.05_ and PPEA_0.1_ at a strain of 50% reached 0.31 and 0.50 MPa, respectively. Compared with the compressive stress of other aerogels that were described in previous studies [30,31], the compressive stresses of the PPEAs were much higher. The high mechanical strengths of the PPEAs might have been because the pectin and PEI were tightly cross-linked and intertwined, forming an orderly and regular three-dimensional network structure, thus effectively improving the mechanical properties of the aerogels.

To determine the wettability of the aerogel, the water contact angles for the PPEAs were observed. As shown in Figure 5B, the water contact angles for the PPEAs were almost undetectable. After dropping one drop of water on the surface of PPEA_0.3_, the water was absorbed immediately, indicating its very good hydrophilicity. The enhanced wettability was because of the high level of hydrophilic groups, e.g., -OH, -COOH, and -NH_2_, that were present in the PPEAs. Moreover, the porous structure with expanded channels may have also facilitated the entrance of water. The excellent water hydrophilicity may have been conducive to the rapid diffusion of heavy metal into the internal network of the aerogels and afforded more available active sites for the adsorption of heavy metal ions [32].

#### 3.2.5. Thermal Stability

In general, an aerogel should be able to withstand a somewhat high temperature, especially when dealing with mining wastewater containing several heavy metals. Any modification of an aerogel should not damage its thermal integrity. The thermal stability of the adsorbents was determined using TGA analysis. The weight loss and corresponding derivative weight curves of the PPEAs are demonstrated in Figure 5C,D, respectively. It was observed that all the samples showed a similar weight loss trend. The mass loss between 50 and 200 °C was supposed to be the vaporization of water from the sample [33]. PPEA_0.05_ had the earliest loss, and the loss was large in the first stage (vaporization of free water) and small in the second stage (vaporization of bound water), while PPEA_0.3_ showed the opposite trend to PPEA_0.05_, which may have been because PPEA_0.3_ had more fine pores (as observed in Figure 4) and better water-holding capacity. The third quick loss stage of 290–360 °C may have been due to the thermal decomposition of PPEAs [34]. In this degradation range, PPEA_0.3_ degraded first, while PPEA_0.05_ degraded most seriously. PPEA_0.1_ and PPEA_0.2_ showed the best thermal stability. It was reported that fast cross-linking kinetics could cause non-homogeneous structures of gels, thus influencing the thermal stability of aerogels [35].

### 3.3. Adsorption Properties of PPEAs

**Figure 6:**
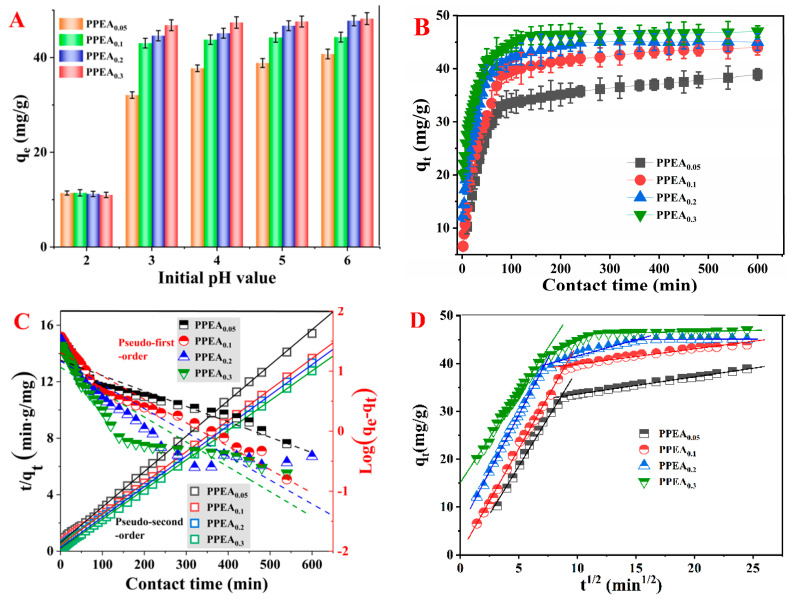

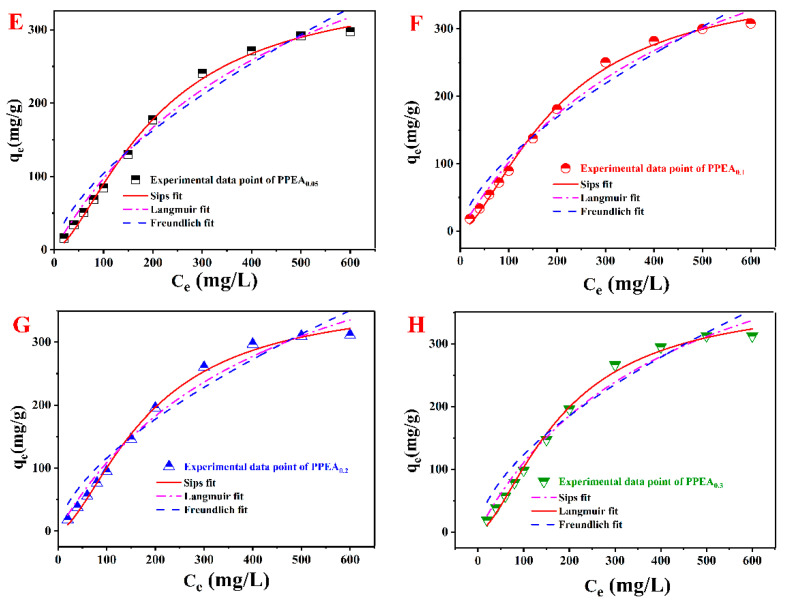
The effect of initial pH (**A**) and contact time (**B**) on the adsorption capacity. The fitting curves with pseudo-first-order and pseudo-second-order kinetic models (**C**), as well as the intra-particle diffusion model (**D**). The adsorption isotherm curves of PPEA_0.05_ (**E**), PPEA_0.1_ (**F**), PPEA_0.2_ (**G**), and PPEA_0.3_ (**H**) as fitted using the Langmuir, Freundlich, and Sips models.

The pH of a lead solution has a critical effect on the adsorption performance of the absorbent as it determines the form of lead ion species and the electrostatic repulsive forces between the absorbents and Pb^2+^ ions. When the pH value is above 6.0, Pb^2+^ will form a precipitate of Pb(OH)_2_, while Pb^2+^ mainly exists in the form of free ions when the pH value is below 6.0. Therefore, the effect of pH on the adsorption of Pb^2+^ was studied at pHs between 2.0 and 6.0. As shown in Figure 6A, the removal rate of Pb^2+^ increased rapidly from pH 2.0 to 3.0, and then reached a plateau when the pH exceeded 3.0. This phenomenon was quite different from the other pectin-based Pb^2+^ adsorbents, which usually showed an optimal adsorption pH of 5.0 [5,36]. The wider pH bearing range of the PPEAs may have been because the co-existence of PEI, which contains a high density of amino groups, changed the adsorption environment. In addition, the ability of aerogels to remove Pb^2+^ was significantly enhanced with the increase in the EGDE content. Both PPEA_0.2_ and PPEA_0.3_ showed strong Pb^2+^ removal efficiency (>90%) in the pH range of 3.0–6.0. These results may have be because the *DM* and the porosity of aerogels became higher with the increase in EGDE content, which facilitated the contact between the active groups and Pb^2+^, thus improving the adsorption efficiency [37,38].

The effect of the contact time on the adsorption of Pb^2+^ was estimated and is shown in Figure 6B. All the PPEAs exhibited a high adsorption rate within the initial 70 min and showed an obvious decrease between 70 and 120 min, after which the adsorption reached a steady state. The high initial adsorption rate could be ascribed to the high concentration gradient and availability of active sites, which acted as a driving force to facilitate the mass transfer from the aqueous media to the PPEA’s surface. However, as the active sites became saturated, the adsorption rate decreased and finally reached a constant level. In addition, the adsorption capacity increased with the amount of EGDE, which was once again attributed to the porous structure of aerogels with abundant functional groups that facilitated the adsorption of Pb^2+^.

To understand the adsorption kinetic behavior, the experimental data were fitted using pseudo-first-order, pseudo-second-order, and intra-particle diffusion models. The corresponding parameters are presented in the Supplementary Materials, Table S1. According to the calculated coefficient of determination (*R*^2^), the pseudo-second-order models exhibited the highest *R*^2^ = 0.9993–0.9998 (Figure 6C). The pseudo-second-order model is based on the assumption that the chemisorption is the rate-limiting step during the adsorption process [39]. The low values of the rate constant (*k*_2_) in the pseudo-second-order model suggested that the adsorption rate was primarily affected by the number of unoccupied functional groups on the adsorbent and the rate of adsorption decreased with the increasing contact time [40,41,42].

According to the SEM, except for abundant functional groups on the PPEAs, the excellent adsorption performance of the PPEAs may be related to their porous characteristics; thus, the experimental results were also fitted using the intra-particle diffusion kinetic model [43]. The intra-particle diffusion model showed two distinct linear stages for PPEA_0.05_ and PPEA_0.1_, but three stages for PPEA_0.2_ and PPEA_0.3_, as illustrated in Figure 6D. Generally, the three stages of the intra-particle diffusion models can be ascribed to (i) the fast external surface adsorption stage, (ii) the slow internal diffusion stage, and (iii) the adsorption equilibrium stage [44]. In the first stage, due to the abundant active sites and the boundary layer effect, Pb^2+^ could be quickly adsorbed onto the adsorbent surface. Subsequently, the diffusion of Pb^2+^ from the adsorbent surface to the pore channel resulted in a decrease in the adsorption rate. Considering that the intra-particle diffusion did not pass through the origin point, as well as the difference in the adsorption rate between the initial and final stages, both the intra-particle diffusion and the surface adsorption were involved in controlling the adsorption rate.

An adsorption isotherm can be used to describe the type of interaction between an adsorbent and heavy metal, which is crucial for optimizing the use of adsorbents. In the present study, adsorption isotherms, namely, nonlinear Langmuir, Freundlich, and Sips models, were chosen to fit the experimental results. The Langmuir isotherm is based on the assumption that the adsorption sites are homogeneous and there is no obvious interaction between the adsorbed substances. In contrast, the Freundlich isotherm describes the adsorption process that occurs on heterogeneous surfaces with different energies [45]. The Sips isotherm is a combination of the Langmuir and Freundlich isotherms. Plots of the fitted isotherms for adsorption of Pb^2+^ are shown in Figure 6E–H, and the adsorption parameters are present in Table S1. The best-fitting equation was based on the highest *R*^2^. According to the fitting results, the Sips isotherm was the most suitable model for describing the adsorption of Pb^2+^ by PPEAs, which means that the adsorption was a monolayer phenomenon and the adsorption sites were heterogeneous. The determined values of q_max_ were 362.95, 375.6, 370.3, and 373.7 mg/g for PPEA_0.05_, PPEA_0.1_, PPEA_0.2_, and PPEA_0.3_, respectively. Comparisons of the q_max_ values presented in this study with those of other pectin-based adsorbents for Pb^2+^ adsorption found that the present PPEAs showed better performance under analogous conditions, as shown in Table 2. The high q_max_ indicated that the PPEAs could be very promising adsorbents for removing Pb^2+^ from the aqueous solutions. However, a promising adsorbent should not only consider the maximum sorption capacity. The other properties (such as mechanical properties, density, and thermal properties) should also be considered. For example, PPEA_0.3_ had the lowest density and best mechanical properties among the PPEAs.

**Figure 7:**
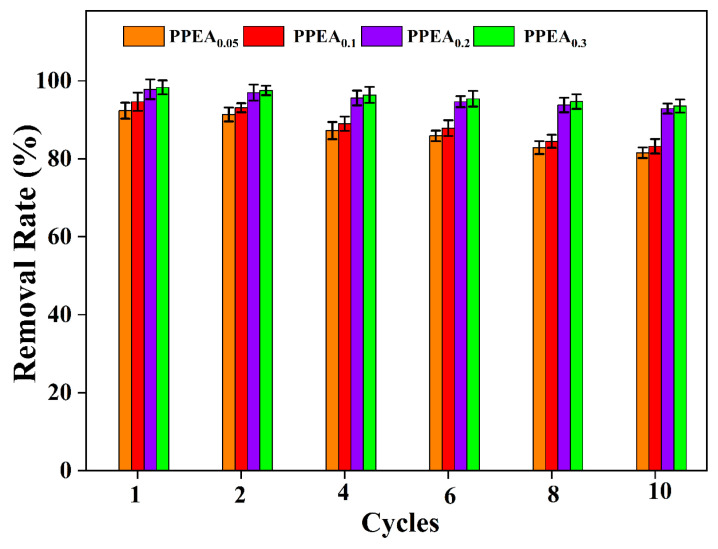
Pb^2+^ removal efficiency of the PPEAs during the adsorption–desorption cycles.

In addition to the excellent mechanical properties and adsorption capacity, good and easy recyclability are also key indexes for developing a new heavy metal adsorbent [52]. In this study, a dilute HNO_3_ solution was used as an eluent to help with the release of Pb^2+^ from the binding sites of the adsorbent, and the PPEAs could be directly salvaged after each adsorption and desorption cycle. The recyclability of the PPEAs was studied through 10 cycles of adsorption–desorption experiments. It was found that PPEA_0.3_ exhibited the best regeneration performance, retaining a removal rate of over 93.5% after 10 cycles (Figure 7). The morphologies of the PPEAs after 10 cycles were examined and are shown in Figure 4B,D,F,H. The structure of the aerogel seemed more compact but kept most of the porous structure. The compact structure might be attributed to the bridging effect of the absorbed Pb^2+^. These Pb^2+^ ions could cross-link the carboxyl group and/or amine groups to produce dense porous networks. The reason that most of the porous structure was retained may have been because the PPEAs had good mechanical stability, which endowed them with excellent adsorption performance in the continuous adsorption–desorption cycles.

### 3.4. Adsorption Mechanism of PPEAs

#### 3.4.1. FTIR after Adsorption

The adsorption mechanism was investigated by comparing the FTIR changes of the PPEAs before and after the adsorption. It was found that the infrared spectroscopy after adsorbing the Pb^2+^ had some obvious variations compared to those before adsorption. A peak around 1382 cm^−1^ was observed for all the PPEAs after adsorption but not for the PPEAs without Pb^2+^. This peak was attributed to the carboxyl groups of pectin that had bound heavy metals [53]. In addition, the peak at ~1645 cm^−1^, which was attributed to asymmetric stretching of the carboxylic C=O double bond, faded. This result confirmed the involvement of carboxyl groups in the binding of Pb^2+^. The broad and wide nature of the peaks (~3200–3400 cm^−1^) of all the modified aerogels was because of mutual conventional hydrogen bonding between abundantly available -OH and -NH functionalities. Because some of these hydrogen bonds were cleaved by the adsorbed Pb^2+^, the separate -NH stretching peak (3255.2 cm^−1^) started to appear in the Pb^2+^ loaded samples [11,54]. The peak at 636.3 cm^−1^, corresponding to the complexation of divalent cation and amine groups [55], was intensified after adsorption. Therefore, the amine groups of the PPEAs were also likely responsible for binding Pb^2+^.

#### 3.4.2. XPS

**Figure 8:**
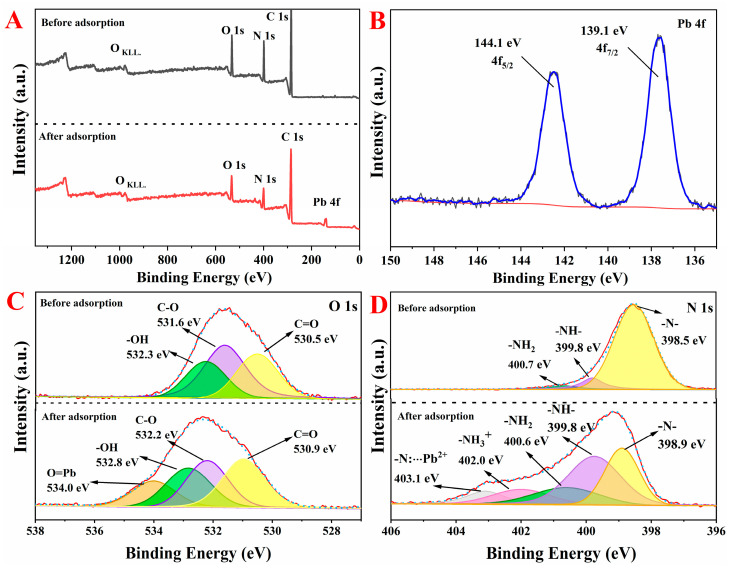
Wide-scan XPS spectra of PPEA_0.1_ before and after the adsorption (**A**), Pb 4f binding energies (**B**), O 1s binding energies (**C**), and N 1s binding energies (**D**).

The XPS wide-scan spectra of PPEAs before and after Pb^2+^ adsorption is shown in Figure 8A. The XPS results of Pb 4f (Figure 8B) showed two energy bands after the adsorption, located at 144.1 and 139.1 eV, corresponding to the binding energy of the Pb 4f_5/2_ and Pb 4f_7/2_ orbitals, respectively. This result showed that Pb^2+^ successfully adsorbed onto the surface of the PPEAs [56]. To further investigate the interactions between the PPEAs and Pb^2+^, the high-resolution O 1s and N 1s spectra before and after the adsorption of Pb^2+^ are shown in Figure 8C,D, respectively. The O 1s spectrum before the adsorption was deconvoluted into three peaks, corresponding to C=O (530.5 eV), C-O (531.6 eV), and -OH (532.3 eV) [57]. After the adsorption of Pb^2+^, it was found that the binding energy of those groups shifted significantly (532.8, 532.2, and 530.9 eV, respectively). Furthermore, a new strong peak appeared at 534.0 eV (O=Pb), indicating that oxygen-containing functional groups, such as -COOH and -OH, had a strong binding ability with Pb^2+^ [58]. In addition, the N 1s peak on the PPEAs was deconvoluted into three characteristic peaks (Figure 8D), where their binding energies were 398.5, 399.8, and 400.7 eV, respectively, corresponding to -N-, -NH-, and -NH_2_, respectively [58,59]. After the adsorption, two new peaks appeared in the N 1s spectra at binding energies of about 402.0 and 403.1 eV. The 402.0 eV represents -NH^3+^, which was formed by combining -NH_2_ with H^+^. The H^+^ was released from the ion exchange between -COOH and Pb^2+^. The new binding energy 403.1 eV appeared after the adsorption, which could be attributed to the formation of a -N···Pb^2+^ complex. According to the theory of hard/soft and acid/base, the presences of donor groups, such as NH^3+^, -NH_2_, and -NH, are responsible for the adsorption of divalent metal cations [60]. Therefore, the XPS spectra of N 1s effectively confirmed that the adsorption mechanisms involved both ion exchange and complexation.

It should be noted that although all the PPEAs showed similar chemical adsorption mechanisms, they showed different adsorption capacities, as we discussed in Section 3.2, which may have been because the adsorption performance of the PPEAs was influenced by two aspects. One was that the donor groups, such as NH^3+^, -NH_2_, and -NH, on the PEI molecular chain and oxygen-containing functional groups, such as -COOH and -OH, on the pectin chain could chelate or complex with Pb^2+^. Second, a highly porous structure is also conducive to the diffusion of heavy metals within the aerogels. The physical and chemical synergistic adsorption resulted in the good adsorption performance of the PPEAs. The adsorption mechanism of the PPEAs’ adsorption of Pb^2+^ is represented by the schematic diagram in Figure 1g.

## 4. Conclusions

Novel 3D pectin-based aerogels (PPEAs) were synthesized by introducing branched PEI onto pectin with EGDE as a cross-linker for the adsorption of Pb^2+^ from an aqueous solution. This material exhibited a high adsorption capacity (>360 mg/g), a porous structure, an ultralight weight (>60 mg/cm^3^), a robust mechanical strength, good thermal stability (~260 °C), and excellent regeneration (>90% removal rate about Pb^2+^ after 10 cycles). The Pb^2+^ adsorption kinetics was controlled by chemisorption, and the Sips model revealed that the adsorption was a monolayer phenomenon and the adsorption sites were heterogeneous. FTIR and XPS indicated that both the amine- and oxygen-containing groups were the main functional adsorbing sites. The newly fabricated pectin-based aerogels are expected to be potential candidates for the efficient, renewable, and recycled adsorption of Pb^2+^-like heavy metals.

## Figures and Tables

**Table 1:** The elemental compositions and degrees of modification (*DM*s) of PPEAs.

Samples	*C* (%)	*N* (%)	*DM* (%)
PPEA_0.05_	40.49 ± 0.06 a	14.98 ± 0.06 a	4.66 ± 0.03 a
PPEA_0.1_	45.80 ± 0.34 b	18.67 ± 0.11 b	6.23 ± 0.03 b
PPEA_0.2_	46.11 ± 0.62 b	19.23 ± 0.29 b	6.74 ± 0.05 c
PPEA_0.3_	46.79 ± 0.07 b	20.21 ± 0.08 c	7.67 ± 0.08 d

Mean values in the same column with different letters are significantly different (Tukey’s test, *p* < 0.05). Data are presented as means ± standard deviations of triplicate measurements.

**Table 2:** Comparison of the adsorption capacities of various pectin-based adsorbents for Pb^2+^ adsorption.

Adsorbents	pH	Temperature (K)	q_max_ (mg/g)	References
Pectin microspheres	5.0	298	325	[46]
Pectin-based polymer hydrogel	5.5	298	130	[47]
Pectin-graft-poly(METAC-co-AMPS)/MMT-C2	-	298	79.78	[48]
Pectin/activated carbon-based porous microsphere	5.0	298	279.33	[49]
Pectin-rich fiber	-	298	184	[50]
HHP-assisted pectinase modified pectin	7.0	298	263.15	[51]
PPEAs	5.0	298	373.7	This study

METAC: 2-(methacryloyloxyethyl) trimethylammonium chloride, AMPS: 2-acrylamido-2-methyl-1-propane sulfonic acid, MMT: composite with montmorillonite, HHP: high hydrostatic pressure. A hyphen in the pH column means no mention was made of the pH in the research.

## Supplementary Materials

The following are available online at https://www.mdpi.com/article/10.3390/foods10123127/s1, Table S1: The parameters of kinetic modeling and isotherm modeling related to the adsorption of Pb^2+^ onto PPEAs.
