# Supplementary material for: Development of Pectin-Based Aerogels with Several Excellent Properties for the Adsorption of Pb2+
Source: Foods. 2021 Dec 16;10(12):3127. doi: 10.3390/foods10123127 (PMC8700957; doi:10.3390/foods10123127)
Supplement: Supplementary file 1 [file foods-10-03127-s001.zip › foods-1465831-supplementary.pdf]

foods

**Supplementary Table**

**Table S1.** The parameters of kinetic modeling and isotherm modeling related to the adsorption of $Pb^{2+}$ onto PPEAs.

| | Model | Equation | Parameters | Values | | | |
|---|---|---|---|---|---|---|---|
| | | | | $PPEA_{0.05}$ | $PPEA_{0.1}$ | $PPEA_{0.2}$ | $PPEA_{0.3}$ |
| Kinetic modeling ($q_t$: the capacity of adsorption at time $t$ (mg/g)) | Pseudo-first order model | $\log(q_e - q_t) = \log q_e - k_1 t$ | $R^2$ | 0.8874 | 0.9261 | 0.8456 | 0.8189 |
| | | $k_1$: the equilibrium rate constant of this model | $k_1$ (×10^{−3}) | 2.570 | 3.900 | 3.970 | 4.150 |
| | Pseudo-second order model | $\frac{t}{q_t} = \frac{1}{k_2 q_e^2} + \frac{t}{q_e}$ | $R^2$ | 0.9993 | 0.9996 | 0.9996 | 0.9998 |
| | | $k_2$: the equilibrium rate constant of this model | $k_2$ (×10^{−3}) | 1.024 | 1.105 | 1.892 | 2.804 |
| | Intra-particle diffusion model | $q_t = k_{ip} t^{1/2} + C_i$ | $R^2$ | 0.7037 | 0.7281 | 0.7047 | 0.6956 |
| | | $k_{ip}$: the rate constant of this model | $k_{ip}$ | 1.038 | 1.494 | 1.2693 | 1.0291 |
| | | $C_i$: the intercept. | $C_i$ | 18.1070 | 16.460 | 22.699 | 28.731 |
| isotherms modeling ($q_e$: the amount of $Pb^{2+}$ adsorpted at equilibrium (mg/g); $C_e$: the equilibrium concentration of $Pb^{2+}$ (mg/L)) | Langmuir model | $q_e = \frac{q_{max} b C_e}{1 + b C_e}$ | $R^2$ | 0.9851 | 0.9856 | 0.9812 | 0.9817 |
| | | | $q_{max}$ | 577.90 | 589.01 | 577.01 | 571.347 |
| | | $q_{max}$: the maximum adsorption capacity (mg/g); b: Langmuir constant. | $b$ | 0.002 | 0.0021 | 0.002 | 0.002 |
| | Freundlich model | $q_e = k_F C_e^n$ | $R^2$ | 0.9929 | 0.9926 | 0.99145 | 0.9538 |
| | | $k_F$: Freundlich constant; | $k_F$ | 5.3677 | 5.7425 | 6.7442 | 8.1738 |
| | | $n$: adsorption intensity | $n$ | 0.6438 | 0.6383 | 0.6174 | 0.5889 |
| | Sips model | $q_e = \frac{q_{max} b_s C_e^n}{1 + b_s C_e^n}$ | $R^2$ | 0.9971 | 0.9972 | 0.9962 | 0.9959 |
| | | | $q_{max}$ | 362.95 | 375.6 | 370.3 | 373.7 |
| | | $B_s$: the Sips equilibrium constant; n: the Sips model exponent | $b_s$ | 2.66 | 2.91 | 2.288 | 2.623 |
| | | | $n$ | 1.55 | 1.53 | 1.605 | 1.581 |
